# Extracellular Vesicles from Follicular and Ampullary Fluid Isolated by Density Gradient Ultracentrifugation Improve Bovine Embryo Development and Quality

**DOI:** 10.3390/ijms22020578

**Published:** 2021-01-08

**Authors:** Anise Asaadi, Nima Azari Dolatabad, Hadi Atashi, Annelies Raes, Petra Van Damme, Michael Hoelker, An Hendrix, Osvaldo Bogado Pascottini, Ann Van Soom, Mojtaba Kafi, Krishna Chaitanya Pavani

**Affiliations:** 1 Department of Reproduction, Obstetrics and Herd Health, Ghent University, 9820 Merelbeke, Belgium; nima.azaridolatabad@ugent.be (N.A.D.); Atashi@shirazu.ac.ir (H.A.); annelrae.raes@ugent.be (A.R.); p.vandamme@ugent.be (P.V.D.); osvaldo.bogado@ugent.be (O.B.P.); Ann.VanSoom@UGent.be (A.V.S.); 2 Department of Animal Reproduction, School of Veterinary Medicine, Shiraz University, Shiraz 7196484334, Iran; kafi@shirazu.ac.ir; 3 Department of Animal Science, Shiraz University, Shiraz 7144165186, Iran; 4 Department of Animal Breeding and Husbandry, University of Bonn, 53012 Bonn, Germany; michael.hoelker@itw.uni-bonn.de; 5 Laboratory of Experimental Cancer Research, Department of Radiation Oncology and Experimental Cancer Research, Ghent University, 9000 Ghent, Belgium; An.Hendrix@UGent.be; 6 Department of Veterinary Sciences, Gamete Research Center, University of Antwerp, 2610 Antwerp, Belgium

**Keywords:** extracellular vesicles, follicular fluid, ampullary oviductal fluid

## Abstract

Extracellular vesicles (EVs) have been isolated from follicular (FF) and ampullary oviduct fluid (AOF), using different isolation methods. However, it is not clear whether different purification methods can affect the functionality of resulting EVs. Here, we compared two methods (OptiPrep™ density gradient ultracentrifugation (ODG UC) and single-step size exclusion chromatography (SEC) (qEV IZON™ single column)) for the isolation of EVs from bovine FF and AOF. Additionally, we evaluated whether the addition of EVs derived either by ODG UC or SEC from FF or AOF during oocyte maturation would yield extra benefits for embryo developmental competence. The characterization of EVs isolated using ODG UC or SEC from FF and AOF did not show any differences in terms of EV sizes (40–400 nm) and concentrations (2.4 ± 0.2 × 10^12^−1.8 ± 0.2 × 10^13^ particles/mL). Blastocyst yield and quality was higher in groups supplemented with EVs isolated from FF and AOF by ODG UC, with higher total cell numbers and a lower apoptotic cell ratio compared with the other groups (*p* < 0.05). Supplementing in vitro maturation media with EVs derived by ODG UC from AOF was beneficial for bovine embryo development and quality.

## 1. Introduction

Over the past decades, assisted reproductive technologies (ARTs) have become an important tool to understand early embryonic development in mammals and can be applied to treat human and animal infertility and to preserve gametes from animals of high genetic merit, such as in endangered species conservation [1]. Despite the great improvement in ARTs in the last decades, conditions of in vitro embryo production are far from the physiological, in vivo conditions [2]. Successful oocyte maturation involves periods wherein the gamete undergoes nuclear, cytoplasmic, and molecular maturation [3]. However, the developmental competence of bovine oocytes to the blastocyst stage under in vitro conditions is limited to around 40%. This may be due to inadequate in vitro maturation conditions that fail to mimic the follicular environment. 

Intercellular communication within the ovarian follicle is crucial to coordinate the development of a competent oocyte that is capable of being fertilized and undergo embryogenesis [4]. Antral follicles in the mammalian ovary are composed of theca, granulosa, and cumulus cells, and the oocyte, and are filled with follicular fluid (FF) [5]. Reciprocal communication between these cells and FF is regulated by endocrine and paracrine signaling factors and results in the exchange of nutrients, antioxidants, and growth molecules, amongst others. This cooperation provides a favorable environment that is vital for the normal development of a fertile oocyte [6]. Recently, extracellular vesicles (EVs) were identified in FF and ampulla oviductal fluid (AOF) [7,8], whereby FF-derived EVs have been described to play a crucial role in follicular development.

Extracellular vesicles are membrane-enclosed vesicles that contain bioactive molecules (i.e., proteins, RNAs, mRNAs, microRNAs (miRNAs), and lipids) and are present in all types of biological fluids [9]. Extracellular vesicles can be classified as exosomes or microvesicles depending on their size, content, and biosynthesis. Exosomes are usually smaller in size (30–150 nm), and they are formed in the late-endosomal compartment by inward budding of the membrane of late multivesicular bodies [10]. Microvesicles are produced by outward budding of the plasma membrane, and they are larger in size (30–1000 nm) compared with exosomes [11]. Exosomes and microvesicles cannot be distinguished with specific markers due to overlap of markers and size. Therefore, EV has been adopted as an inclusive term that encompasses both kinds of vesicles outside the plasma membrane [12].

Ever since it has been discovered that FF-derived EVs may contain miRNA and that they can be incorporated by granulosa cells in vitro [7,13], there is a growing research interest in the isolation of EVs from FF of different-sized follicles and their supplementation during in vitro oocyte maturation. This approach resulted in the improvement of cumulus expansion and the modification of cumulus expansion-related transcripts in bovine cumulus cells [14,15,16,17,18]. However, the cargo of FF-derived EVs is greatly determined by the pathophysiological state of the cells where they originate from. Both the physiological state of the animal and the stage of the estrous cycle are known to influence the content of the EVs [18,19].

Recent studies have demonstrated that ampulla oviductal epithelial cells co-incubated with cumulus-oocyte complexes (COCs) improved the subsequent embryo development [20,21]. After ovulation, oocyte maturation can continue in the ampullary region of the oviduct [22]. Based on these observations, we hypothesized that the EVs isolated from ampulla oviductal fluid (AOF) may be used as a supplement during the final stages of bovine oocyte maturation. To date, most of the studies performing functionality testing of EVs derived from FF have been using the “gold standard” ultracentrifugation isolation protocol [7,14,16,17,18]. However, it has been demonstrated that using high-speed ultracentrifugation can damage the EVs due to shear forces [12,23,24,25]. OptiPrep^TM^ density gradient ultracentrifugation is a reliable isolation technique to purify EVs without contamination of other nanoparticles (high-density lipoproteins and ribonucleoproteins) [23,25]. Additionally, size exclusion chromatography may be a useful alternative to obtain a higher functionality of purified EVs [26]. The main objective of the present study was to determine whether FF and AOF-derived EVs applied during in vitro maturation could affect the subsequent embryo development. We also aimed to investigate which of two different isolation techniques (OptiPrep^TM^ Density Gradient Ultracentrifuge or qEV IZON^TM^ single column) was the most efficient for obtaining functional EVs.

## 2. Results

### 2.1. Equivalent Efficiency of OptiPrep™ Density Gradient Ultracentrifugation and Single-Step Size Exclusion Chromatography (qEV IZON™ Single Column) in Isolating Extracellular Vesicles

**Figure 1:**
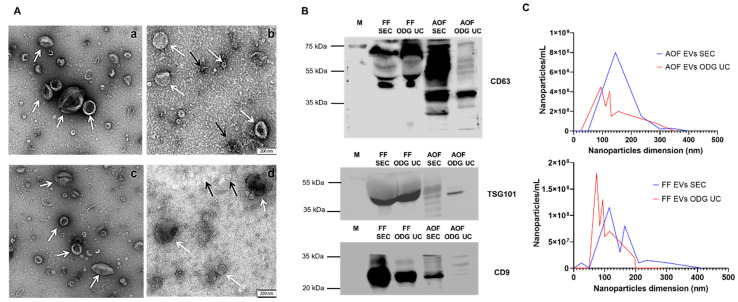
Characterization of extracellular vesicles (EVs) isolated from follicular fluid (FF) and ampullary oviductal fluid (AOF) by two different EV isolation methods: OptiPrep^TM^ density gradient ultracentrifugation (ODG UC) and single-step size exclusion chromatography (SEC) (qEV IZON™ single column)). (**A**) Transmission electron microscopy showing morphological structures of EVs (white arrows) and protein aggregates (black arrows) from AOF and FF by the ODG UC (a,c) and SEC (b,d) isolation methods, with sizes ranging from 40 nm to 400 nm in diameter (scale bar: 200 nm). (**B**) Western blotting analysis showed the presence of EV-associated proteins CD63 (42 kDa), CD9 (25 kDa), and TSG101 (42 kDa) in AOF and FF by both isolation protocols (ODG UC and SEC). (**C**) Analysis of the particle size (nm) distribution of EVs isolated from AOF and FF by ODG UC (Red) and SEC (Blue) is determined by a nanoparticle tracking analysis (NTA). Particles ranged between 50 to 400 nm. Abbreviations: M = marker (protein ladder).

The characterization of EVs isolated from AOF and FF by both OptiPrep™ density gradient ultracentrifugation (ODG UC) and size exclusion chromatography (SEC) demonstrated equal efficacy. Firstly, a transmission electron microscopy (TEM) analysis confirmed the presence of EVs of approximately 40 to 400 nm in size in both fluids (FF and AOF) with both isolation techniques (ODG UC and SEC) (Figure 1). Extracellular vesicles isolated from FF and AOF by SEC (Figure 1A (b,d)) presented protein aggregates (indicated with black arrows). Most EV structures in FF and AOF were cup-shaped, and a few were spherical, with sizes ranging from 40 to 400 nm (Figure 1C). Similarly, a nanoparticle tracking analysis (NTA) of the FF and AOF samples demonstrated that the particle size ranged between 50 to 400 nm. The EV particle concentration in the FF EV samples was in a similar range for both EV isolation methods, as the particle concentration of FF EVs isolated by ODG UC and SEC was 2.4 ± 0.2 × 10^12^ particles/mL and 7.2 ± 0.79 × 10^12^ particles/mL, respectively (as detailed in Table 1). The mean, mode, and median particle sizes were lower in the FF EVs isolated by ODG UC vs. FF EVs isolated by SEC (Table 1). For AOF-derived EVs, the particle concentration was much higher when ODG UC isolation was performed (1.8 ± 0.2 × 10^13^ particles/mL) compared to SEC isolation (6.4 ± 0.5 × 10^12^ particles/mL), whereas the mean, mode, and median particle sizes were within a similar range (as detailed in Table 1). Western blot analysis identified EV-associated proteins (CD63, CD9, and TSG101) in the EVs derived from both biological fluids (FF and AOF) isolated by the ODG UC and SEC methods (Figure 1B). Extracellular vesicles isolated by ODG UC showed relatively light band formation in all the three EV specific proteins (CD63, CD9, and TSG101) compared with EVs isolated by the SEC method. This provides additional proof that EVs isolated by SEC contain more proteins than with ODG UC. This is also visible in the TEM images (Figure 1A), where some protein aggregates were observed in EVs derived by the SEC method.

### 2.2. Higher Functionality of Extracellular Vesicles Isolated Using OptiPrep™ Density Gradient Ultracentrifugation Compared to Single-Step Size Exclusion Chromatography (qEV IZON™ Single Column)

#### 2.2.1. Embryo Development 

The cleavage rate and blastocyst yield were used to evaluate the effects of EVs from two different sources (FF and AOF) and isolated by two different techniques (ODG UC and SEC) on embryo development (Table 2). Although there were no differences in the cleavage rates between the groups, the blastocyst yield was affected by the treatment (*p* < 0.05). The blastocyst yield was higher in the groups of FF and/or AOF EVs isolated by ODG UC (*p* < 0.05) when compared to groups supplemented with FF and/or AOF EVs isolated by SEC and compared to the control groups. There were no significant differences between FF and/or AOF EVs isolated by SEC and the control groups (control and negative control), except for AOF EVs isolated by SEC, which had a higher blastocyst yield than the control group (*p* < 0.05). For both isolation methods (ODG UC and SEC), there was no difference in blastocyst yield when EVs were derived from FF, AOF, or the combination of both (*p* > 0.05).

#### 2.2.2. Embryo Quality

**Figure 2:**
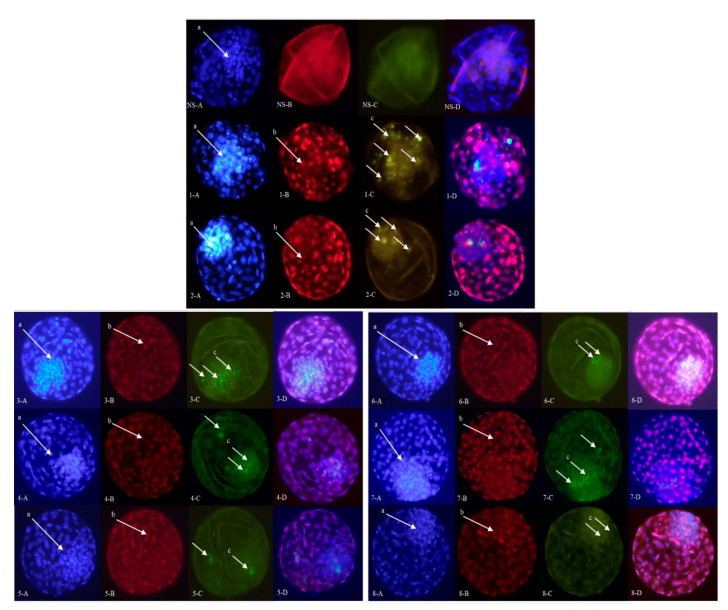
Differential apoptotic staining of the expanded blastocysts (eight groups) (A–D). Representative blastocysts of all groups (1–8) are presented (see Experimental Design of the Materials and Methods and Figure 4 for details). NS: negative staining control. Both the trophectoderm (TE) and inner cell mass (ICM) (arrow a) nuclei were stained with Hoechst (1.A–8.A). The CDX2 antibody (labeled with Texas Red) resulted in a red fluorescent signal in TE cells (arrow b) (1.B–8.B). Apoptotic cells (arrow c) showed a green fluorescent signal (anti-caspase 3 antibody labeled with FITC) (1.C–8.C). Merged pictures (1.D–8.D) (40× magnification).

The assessment of the embryo quality was performed by using differential apoptotic staining of expanded blastocysts (Figure 2). The effects of experimental treatments on the apoptotic differential cell counts are presented in Table 3. The average numbers for the inner cell mass (ICM), trophectoderm (TE), and total cell number (TCN) were lower in the control groups compared to the different treatment groups (*p* < 0.05) (Table 3). Additionally, the apoptotic cell ratio (ACR) was higher in the control groups compared to the other groups (*p* < 0.05) (Table 3). When FF and/or AOF EVs were supplemented after ODG UC isolation, the average numbers for the TCN, ICM, and TE were higher than those in the corresponding SEC groups (*p* < 0.05) (Table 3). Additionally, the ACR was lower in the ODG UC groups than that in the corresponding SEC groups (*p* < 0.05), except for the FF AOF SEC group (*p* ≥ 0.05) (Table 3).

## 3. Discussion

There is growing evidence that EVs isolated from follicular and oviductal fluids are involved in different biological functions related to follicular growth, oocyte maturation, and embryo development [14,15,16,17,18]. However, no study has demonstrated that purification methods for EVs can exert a different functional impact on bovine oocyte maturation and subsequent embryo development. Our study demonstrated that the ODG UC EV isolation method yielded EVs with a higher purity, which had a significantly better functional impact on embryo development and quality compared to EVs isolated by single-step size exclusion chromatography using qEV IZON™ single column. As per our knowledge, this is the first study to demonstrate that AOF-derived EVs can improve subsequent bovine embryo development and quality. Mimicking the in vivo process of ovulation and oocyte maturation had a significant impact on embryo development and quality, which was achieved by supplementing EVs derived from AOF to in vitro maturation (IVM) medium during the last 4.5 h of oocyte maturation in this study.

This study demonstrated that ODG UC supersedes SEC using qEV IZON™ single column in terms of EV purity and functionality preservation. It was previously demonstrated by our group [12,23,25] that ODG UC is superior in conserving EV-associated proteins and RNAs compared with other standard multistep ultracentrifugation protocols and the SEC method. In the current study, we identified that SEC (qEV IZON™ single column) resulted in some protein aggregates (possibly the lipoproteins). These findings are in agreement with our previous study [23], in which EVs were isolated from a medium conditioned by bovine embryos by means of SEC but still showed lipoprotein contamination. Similarly, Takov et al. [27] demonstrated that lipoproteins were co-isolated with EVs derived from plasma when SEC is applied. The presence of these protein aggregates in SEC-derived EVs might be the possible factor for decreased embryo development and quality compared with the ODG UC-derived EV treatment group.

In the current study, EVs were isolated by applying OGD UC and SEC, since these techniques provide proper vesicle structure, and the integrity of EVs remains largely intact [23]. Standard differential ultracentrifugation yields lower concentrations of EVs, and EVs can be damaged due to different applied shear forces [25]. Most of the research that has been published recently on EVs derived from reproductive fluids has been using differential ultracentrifugation protocols [8,14,28,29]. Although the purity of post-ultracentrifugation EVs is still under debate, concerns are arising mainly because of the residual background materials observed under TEM. Besides, it was previously demonstrated by our group [23] that, after differential ultracentrifugation, EVs can form aggregates after pelleting. Moreover, non-EV-specific markers proteins (ApoA-I and AGO-2) have been identified in the EV pellet sample.

Our study showed that the supplementation of EVs derived from AOF to IVM medium for the final 4.5 h of maturation considerably increased embryo development compared to FF EV supplementation to IVM for the first 18 h of IVM (for ODG: 48.74 vs. 45.41% for AOF vs. FF EVs and, for SEC: 39.79 vs. 35.85% for AOF vs. FF EVs, respectively), even though it was not statistically significant. These results partly support our hypothesis that the final part of oocyte maturation is continued to the ampullary region of the oviduct. It is known that the three oviductal regions: infundibulum, isthmus, and ampulla play a critical role in gamete physiology, fertilization, and preimplantation embryo development. Particularly, the ampullary region of the oviduct is associated with hardening of the zona pellucida [22]. It has been demonstrated that epithelial cells harvested from the ampullary segment of the ovine oviduct induce ZP hardening in vitro [30]. It was reported that coincubation of the ovine oocyte with ampulla oviductal epithelial cells obtained from adult sheep during the 24-h IVM improved the oocyte competence and subsequent in vitro embryo production efficiency [21]. Lee et al. [31] showed that a co-culture with canine oviductal cells improved in vitro porcine oocyte maturation and, also, embryo developmental competence. Recently, it was also demonstrated that co-culturing bovine oocytes with a monolayer of frozen–thawed bovine ampullary cells during the last six h of IVM increased both the blastocyst yield and quality of the subsequent embryos [20]. Notably, in support of our data, supplementing oviductal EVs had a positive impact on the IVM of canine oocytes [32]. This study also demonstrated that canine COCs can uptake oviductal EVs, showing that cumulus cells are involved in paracrine mechanisms as a bridge between the oviduct and oocytes.

In conclusion, this is the first study to show that EVs from in vivo-derived FF exert a positive impact on embryo development in cattle. Moreover, we also observed a beneficial effect of AOF EVs when added during the final hours of maturation. The superior method for the isolation of purified EVs with excellent function from biological fluids was the ODG UC method [25]. These results highlight the potential of EVs derived from biological fluids for addition to culture media to mimic in vivo maturation and can open new perspectives for the optimization of assisted reproductive technologies by improving in vitro oocyte maturation in cattle and other species, like equine, canine, feline, and wildlife species.

## 4. Materials and Methods

### 4.1. Media and Reagents

Tissue culture media (TCM)-199 medium, minimal essential medium (MEM), nonessential amino acids (100×), synthetic basal medium eagle’s amino acids, gentamycin, and kanamycin were purchased from Life Technologies Europe (Ghent, Belgium). Phosphate-buffered saline (PBS) was obtained from Gibco™ 20012019, Thermo Fisher Scientific (Waltham, MA, USA). All other chemicals not otherwise listed were obtained from Sigma-Aldrich (Diegem, Belgium). All media were filtered before use (0.22 µM; GE Healthcare—Whatman, Diegem, Belgium).

### 4.2. Animal Handling, Bovine Follicular, and Ampullary Oviductal Fluid Collection 

Animal handling was carried out in accordance with the German Law of Protection (TierSchG & TierSchVersV). Experimental protocols performed on cows in this study were approved by the state office for Nature, Environment, and Consumer Protection of North Rhine-Westphalia, Germany (Landesamt für Natur, Umwelt und Verbraucherschutz Nordrhein-Westfalen, Deutschland). The blood sample collection and ovum pick-up (OPU) procedures were approved under license numbers 84-02.04.2015.A139 and 84-02.04.2014.A500, respectively.

**Figure 3:**
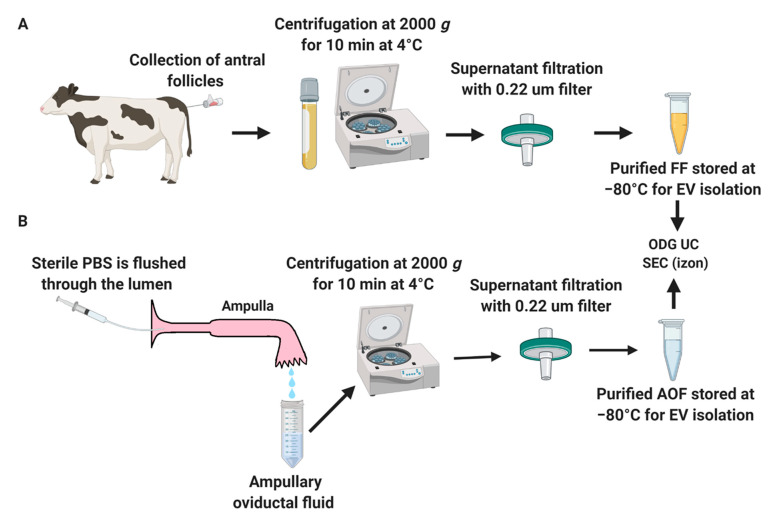
Schematic representation of follicular fluid (FF) (**A**) and ampullary oviductal fluid (AOF) (**B**) collection. Follicular fluid was isolated from estrous Holstein heifers with antral follicle sizes larger than 12 mm. Ampullary fluid was flushed from the oviduct at the early postovulatory phase of the estrous cycle. Both FF and AOF fluids were subjected to EVs isolation methods OptiPrep™ density gradient ultracentrifugation (ODG UC) and single-step size exclusion chromatography (SEC) (qEV IZON™ single column). PBS: phosphate-buffered saline.

Follicular fluid (FF) was collected from four Holstein heifers. Their ages were 15–18 months at the time of follicular fluid collection. Animals were synchronized according to the following schedule: Synchronization was performed by the administration of 500 mg of cloprostenol, i.e., Estrumate. This treatment was repeated after 11 days. Forty-eight hours after each of the cloprostenol treatments, heifers received 0.02 mg of GnRH (Receptal). Ovum pick-up was performed 20–22 h after the final GnRH injection only from follicles with diameters > 12 mm. The FF samples were centrifuged at 2000× *g* at 4 °C for 10 min, followed by filtration through a 0.22-µm filter to remove cells and debris (as detailed in Figure 3A). The FF samples were stored at −80 °C until further analysis. 

Oviducts and ovaries from slaughtered cows that were at the early postovulatory phase of the estrous cycle were selected, based on the presence of a corpus hemorrhagicum [33]. The oviducts and ovaries were transported to the laboratory in ice pack containers within two hours after collection. 

Connective tissue surrounding the oviducts were removed with a scalpel blade, and oviducts were washed twice in normal saline solution and in 70% ethanol solution. Then, oviducts were trimmed, washed with PBS (containing 50-μg/mL kanamycin), and stored in PBS at 4 °C until further utilization. 

Next, the ampulla was separated at the ampullary–isthmic junction, which was identified by a marked reduction in the size of the oviductal diameter [8]. Then, the ampullary lumen was flushed gently with 2-mL sterile PBS using a 22 or 24-G catheter. The AOF samples were centrifuged at 2000× *g* at 4 °C for 10 min, followed by filtration through a 0.22-µm filter to remove all oviductal epithelial cells and debris (as detailed in Figure 3B). To increase the concentration of the AOF samples, 2 mL of pooled AOF were loaded in Amicon^®^ Ultra-2 10-k centrifugal filters (UFC201024, Merck Millipore, Billerica, MA, USA). Around 200-µL AOF was retrieved from the flowthrough reservoir [12]. Next, the AOF samples were stored at −80 °C until further analysis

### 4.3. Extracellular Vesicles Isolation by OptiPrep^TM^ Density Gradient Ultracentrifuge

Extracellular vesicles isolated from FF and AOF were obtained via ODG UC [25]. Briefly, appropriate amounts of homogenization buffer (10-mM Tris-HCl, 1-mM EDTA, and 0.25-M sucrose (pH 7.4)) and iodixanol working solution were mixed to prepare gradients of 5%, 10%, 20%, and 40% iodixanol solutions. The iodixanol working solution was made by adding a solution buffer (60-mM Tris-HCl, 6-mM EDTA, and 0.25-M sucrose (pH 7.4)) to a stock solution of OptiPrep™ (60% (*w/v*) aqueous iodixanol solution). The gradient was prepared in a 16.8-mL open-top polyallomer tube (Beckman Coulter, Brea, CA, USA) by layering 4 mL of 40%, 4 mL of 20%, 4 mL of 10%, and 3.5 mL of 5% solutions on top of each other. The FF or AOF were overlaid onto the top of the gradient. Subsequently, the gradient was centrifuged at 4 °C for 18 h at 100,000× *g* (SW 32.1 Ti rotor, Beckman Coulter, Brea, CA, USA). Out of 16 layers of gradient fractions, EVs were mostly found in fractions 8 and 9 [25]; both fractions were pooled and diluted in 14-mL PBS and, subsequently, centrifuged for 3 h at 100,000× *g* and 4 °C. The resulting pellet was resuspended in 100-µL PBS and stored at −80 °C for further quantification and identification.

### 4.4. Extracellular Vesicles Isolation by Single-Step Size Exclusion Chromatography 

Extracellular vesicles isolated from FF and AOF were also obtained via single-step size exclusion chromatography (qEV IZON™ single column) (Cat. Number: qEV single ser. Y1000588, Oxford, UK). The IZON™ column was washed with PBS (pH: 7.4, 0.22-µm filtered). A maximum of 200 µL of FF or AOF was loaded onto the column, followed by elution with 5-mL freshly filtered PBS (pH: 7.4, 0.22-µm filtered). As per the manufacturer’s instructions, EV-rich fractions 5 to 15 were pooled and loaded in Amicon^®^ Ultra-2 10-k centrifugal filters (UFC201024, Merck Millipore, Billerica, MA, USA) and centrifuged at 4 °C for 40 min at 3000× *g* using a swinging bucket rotor. The concentration of the samples was achieved by upside-down centrifugation at 4 °C for 2 min at 1000× *g* [12]. Around 200 µL of the EVs were retrieved from the flowthrough reservoir and stored at −80 °C until further analysis.

### 4.5. Characterization of Extracellular Vesicles

#### 4.5.1. Transmission Electron Microscopy

Extracellular vesicles from FF and AOF isolated by two different techniques, ODG UC and SEC, were subjected to transmission electron microscopy (TEM). Briefly, EVs were fixed overnight (2% glutaraldehyde and 0.1-M cacodylate buffer) and washed with 0.1-M sodium cacodylate buffer. Each sample was deposited on precoated formvar/carbon support film copper mesh electron microscopy grids (FCF200H-CU-TB; Aurion, Leiden, The Netherlands). Each grid was rinsed and filtered with double-distilled water, then stained for 45 s with 1% uranyl acetate. All samples were examined using electron microscopy (JEM 1400 plus, JEOL, Benelux, Brussels, Belgium). Images were made by a Quemasa charge-coupled device camera (Olympus Soft Imaging solutions GMBH, Munster, Germany).

#### 4.5.2. Nanoparticle Tracking Analysis

To determine the particle size and concentration of EVs from FF and AOF (isolated using both ODG UC and SEC), nanoparticle tracking analysis (NTA) was performed with a NanoSight LM10 microscope (Malvern Instruments Ltd., Malvern, UK). Briefly, extracellular vesicles were diluted in PBS to achieve a concentration between 3 × 10^8^ to 1 × 10^9^ particles/mL. The diluted sample was mixed by vortexing and injected into the measurement chamber. For each sample, three individual videos of 60 s were recorded. Triplicates of the same dilution were performed, and the average was analyzed with detection threshold 3 and camera level 13. All videos were analyzed by NTA Software version 3.2.

#### 4.5.3. Western Blotting 

To test the presence of EVs and other nanoparticles in all samples, Western blotting (WB) was performed with EV-specific (CD63, CD9, and TSG101) markers. All samples were suspended in a reducing buffer (0.005% bromophenol blue, 3% 2-mercaptoethanol, 9.2% SDS, 40% glycerol, and 0.5-M Tris-HCl (pH 6.8)) and boiled for 5 min at 95 °C. Protein samples were separated by SDS polyacrylamide gel electrophoresis and, subsequently, transferred to a nitrocellulose membrane (Bio-Rad, Hercules, CA, USA). Next, the membrane was blocked at room temperature with 5% BSA + 0.5% Tween for 45 min. Subsequently, the membranes were exposed to CD63 rabbit (1:250 in 5% BSA + 0.5% Tween PBS ab68418, Abcam, Cambridge, UK), TSG101 mouse (1:1000 in 5% BSA + 0.5% Tween PBS, sc-7964, Santa Cruz, Oregon, CA, USA), and CD9 rabbit (1:1000 in 5% BSA + 0.5% Tween PBS, CST-D3H4P, Cell Signaling Technology, Boston, MA, USA) primary antibodies at 4 °C. After overnight incubation, the membranes were extensively washed with 0.5% Tween in PBS. Then, the membranes were incubated with the appropriate secondary antibodies (anti-mouse immunoglobulin G (IgG) (GE Healthcare, Buckinghamshire, UK), 1:3000 in 5% BSA + 0.5% Tween PBS and anti-rabbit IgG (GE Healthcare, Buckinghamshire, UK), 1:4000 in 5% BSA + 0.5% Tween PBS). After the final washing step, chemiluminescence substrate (Western Bright Sirius, Advansta, Menlo Park, CA, USA) was added to the membranes. Imaging was performed using Proxima 2850 Imager (IsoGen Life Sciences, De Meern, The Netherlands). 

#### 4.5.4. EV TRACK

All relevant data of the performed experiments were submitted to the EV-TRACK knowledgebase (EV-TRACK ID: EV200013) [34].

### 4.6. In Vitro Embryo Production 

**Figure 4:**
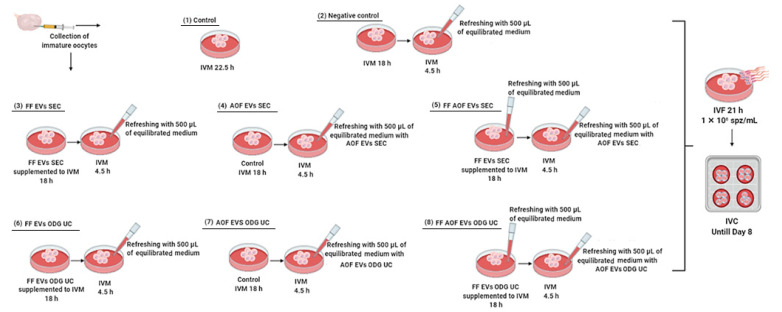
Schematic overview of the experimental design providing the in vitro maturation (IVM) details, by supplementing IVM medium with extracellular vesicles (EVs) derived from follicular fluid (FF) (with a protein concentration of 12.5 µg/mL) and ampullary oviductal fluid (AOF) (with a protein concentration of 1.7 µg/mL) by two different isolation protocols: single-step size exclusion chromatography (SEC) (qEV IZON™ single column) and OptiPrep™ density gradient ultracentrifugation (ODG UC). Cumulus-oocyte complexes (COCs) were distributed into eight different groups for maturation, followed by in vitro fertilization (IVF) and in vitro culture until day 8. Follicular fluid-derived EVs either by the qEV IZON^TM^ single column method or OptiPrep^TM^ density gradient ultracentrifugation method are represented as FF EVs SEC and FF EVs ODG UC, respectively. Ampullary oviductal fluid-derived extracellular vesicles, either by the qEV IZON™ single column method or OptiPrep^TM^ density gradient ultracentrifugation method, are referred to as AOF EVs SEC and AOF EVs ODG UC, respectively.

Routine in vitro methods were used for bovine embryo production as described by Wydooghe et al. [35]. Briefly, bovine ovaries were transported from the local slaughterhouse to the laboratory and processed within 2 h after collection. The ovaries were washed in warm physiological saline with kanamycin (25 mg/mL) and then sterilized with 95% alcohol. Cumulus-oocyte complexes were collected by aspirating 2–8-mm-diameter follicles using an 18-gauge needle attached to a 10-mL syringe. Uniformly granulated cytoplasms surrounded by at least 3 compact layers of cumulus cells were selected and washed in warm equilibrated wash medium (HEPES-buffered TCM-199 and 50-μg/mL gentamicin). Depending on the experimental set-up (as detailed in Figure 4), the COCs were divided into eight groups: (1) control, (2) negative control, (3) follicular fluid-derived EVs by single-step size exclusion chromatography (FF EVs SEC), (4) ampullary oviductal fluid-derived EVs by single-step size exclusion chromatography (AOF EVs SEC), (5) FF EVs SEC + AOF EVs SEC, (6) follicular fluid-derived EVs by OptiPrep™ density gradient ultracentrifugation (FF EVs ODG UC), (7) ampullary oviductal fluid-derived EVs by OptiPrep™ density gradient ultracentrifugation (AOF EVs ODG UC), and (8) FF EVs ODG UC + AOF EVs ODG UC. Each group consisted of 55–60 COCs and were matured in 500-μL maturation medium (modified bicarbonate buffered TCM-199 supplemented with 50-μg/mL gentamicin and 20-ng/mL epidermal growth factor) either supplemented with EVs or not at 38.5 °C in 5% CO_2_ in humidified air for 22.5 h (control group) or for 18 h + 4.5 h (the remaining groups).

Sperm separation and selection were performed using a discontinuous 45%/90% Percoll^®^ gradient (GE Healthcare Biosciences, Uppsala, Sweden) [35]. Briefly, frozen–thawed semen straws from a bull with proven field fertility were placed on top of the 45%/90% Percoll^®^ gradient. After centrifuging twice, the sperm pellet was collected and washed in IVF—Tyrode’s albumin lactate pyruvate medium (TALP) medium, containing a bicarbonate-buffered Tyrode solution. The sperm concentration was determined and adjusted to a final concentration of 1 × 106 spermatozoa/mL using IVF–TALP medium enriched with BSA (Sigma A8806; 6 mg/mL) and heparin (25 mg/mL). After 22.5 h of in vitro maturation, oocytes were washed in 500-µL IVF—TALP and subsequently incubated in 500 μL IVF—TALP with spermatozoa for 21 h at 38.5 °C in 5% CO_2_ in humidified air. 

Presumptive zygotes were denuded 21 h post-insemination (hpi) and cultured in groups of 30 in 50-µL droplets of synthetic oviductal fluid enriched with nonessential and essential amino acids (SOFaa); 4-mg/mL BSA; and ITS (5-mg/mL insulin, 5-mg/mL transferrin, and 5-ng/mL selenium). Each medium droplet was covered with paraffin oil and incubated at 38.5 °C in 5% CO_2_, 5% O_2_, and 90% N2 for 8 days. 

Follicular fluid-derived EVs either by single-step size exclusion chromatography (qEV IZON™ single column™) or OptiPrep™ density gradient ultracentrifugation will be further referred to as FF EVs SEC and FF EVs ODG UC, respectively. Similarly, AOF-derived EVs either by single-step size exclusion chromatography (qEV IZON™ single column) or OptiPrep™ density gradient ultracentrifugation will be referred to as AOF EVs SEC and AOF EVs ODG UC, respectively. 

### 4.7. Assessment of Embryo Development and Quality

Embryo development was determined by the cleavage rate (defined as the total number of cleaved zygotes/total number of fertilized oocytes) and blastocyst yield (defined as the total number of blastocysts/total number of fertilized oocytes). The cleavage rate was recorded on day 2 (48 hpi), and the blastocyst yield was recorded 8 days post-insemination (dpi).

Differential apoptotic staining of expanded blastocysts was performed using the procedure, as described by Wydooghe et al. [36]. Briefly, at 8 dpi, blastocysts were fixed for 20 min in 2% paraformaldehyde (*w/v*). Subsequently, the embryo quality, assessed by differential apoptotic staining, was scored by double-immunofluorescent staining against CDX2 and active caspase-3. CDX2 is a transcription factor that is only expressed in the cells of the trophectoderm, while active caspase-3 has a central role in the apoptotic pathway. Moreover, Hoechst 33342 staining was used to stain all the nuclei of the embryos. In order to adjust the immunofluorescent experimental data, a negative control staining group was used, in which CDX2 and active caspase-3 antibody were omitted from the procedure. This staining protocol allowed the evaluation of three crucial parameters of embryo quality, i.e., the total cell number (TCN), the proportion of inner cell mass (ICM) relative to the TCN (ICM ratio), and the apoptotic cell ratio (ACR), which is the percentage of apoptotic cells relative to the TCN.

### 4.8. Experimental Design 

#### 4.8.1. Quality and Efficiency Assessment of Two Methods for Isolation of Extracellular Vesicles

As we demonstrated before, the ODG UC method is efficient for isolating EVs without contaminants [12]. However, it is a time-consuming protocol [25] and may cause mechanical damage to the EVs [24]. Recently, a single-step size exclusion chromatography method (qEV IZON™ single column) was used to isolate EVs in a shorter time span with higher efficiency [37]. To examine which one of both isolation protocols was the most efficient for the isolation of functional EVs, two different biological fluids, FF and AOF (as detailed in Figure 4), were used in the current study. As detailed above, the EVs were isolated by using ODG UC and IZON^TM^ from the FF and AOF samples, and all the EV characterization techniques were performed. 

#### 4.8.2. Assessing Functionality of Extracellular Vesicles Derived from Follicular and Ampullary Oviductal Fluid during In Vitro Maturation

The presence of EVs in FF and AOF was firstly confirmed by Western blot, and the protein concentrations of the isolated EV samples (FF EVs SEC, FF EVs ODG UC, AOF EVs SEC, and AOF EVs ODG UC) were measured by Nanodrop™ (ND-100 spectrophotometer A280 nm, Wilmington, DE, USA). Maturation of bovine oocytes in situ is initialized in the follicles and finalized in the ampullar region of the oviducts for 3 to 6 h post-ovulation. Based on this in vivo situation, the cumulus-oocyte complexes (COCs; n = 1740, 4 replicates) were allocated into 8 different groups (as shown in Figure 4). 

We designed the experiment to mimic the in vivo situation as much as possible. In the control IVM condition, COCs matured for 22.5 h in standard IVM medium (i.e., without EV supplementation) (Figure 4(1)), and for the negative control (Figure 4(2)), COCs were cultured in IVM medium for 18 h. After 18 h, the IVM medium was refreshed with 500 µL of fresh equilibrated IVM medium, and COCs were cultured for the remaining 4.5 h. For the next two treatments, FF EVs isolated either by SEC or ODG UC (Figure 4(3,6)) were supplemented with the IVM medium with a protein concentration of 12.5 µg/mL. After 18 h, the IVM medium was refreshed with 500 µL of fresh equilibrated IVM medium for the remaining 4.5 h. For the next two treatments, COCs were cultured in standard IVM medium for the first 18 h. After 18 h, the medium was refreshed with 500 µL of a double concentration of AOF EV (3.4 µg/mL) medium, isolated either by SEC or ODG UC, to obtain a final concentration of 1.7 µg/mL for the remaining 4.5 h of IVM (Figure 4(4,7)). For the last two treatments, FF EVs isolated either by SEC or ODG UC (Figure 4(5,8)) were supplemented with an EV protein concentration of 12.5 µg/mL to the IVM medium for the first 18 h. After 18 h, the medium was refreshed with 500 µL of fresh equilibrated maturation medium. For the remaining 4.5 h, the medium was refreshed with 500 µL of a double concentration of AOF EVs (3.4 µg/mL) isolated either by the SEC or ODG UC protocol to have a final concentration of 1.7 µg/mL. All treatment groups were subjected to in vitro fertilization, followed by in vitro culture. On 8 dpi, the blastocyst development was evaluated for all treatment groups, while the embryo quality was assessed by differential apoptotic staining of the expanded blastocysts. 

### 4.9. Statistical Analysis

The effect of EV supplementation during oocyte IVM on the cleavage rate; blastocyst yield; embryo cell number (ICM, TE, and ICM/TE ratio); and apoptotic cell ratio was analyzed using one-way analysis of variance (ANOVA) in R (R Core Team, 2019). The assumptions of normality and homogeneity of the residual variances were evaluated using the Shapiro–Wilk and Bartlett tests, respectively. The means of the groups were compared at a significance level of *p* < 0.05. 

## Figures and Tables

**Table 1:** Comparison of extracellular vesicle isolation methods by nanoparticle analysis.

Source of EVs	Follicular Fluid		Ampullary Oviductal Fluid	
Isolation Technique	SEC	ODG UC	SEC	ODG UC
Mean	136.7 ± 3.9 nm	107.9 ± 5.4 nm	165.5 ± 5.8 nm	166.9 ± 9.7 nm
Mode	109.1 ± 2.5 nm	79.9 ± 6.5 nm	128.1 ± 14.3 nm	145.5 ± 5.3 nm
SD	52.1 ± 4.6 nm	47.8 ± 5.9 nm	84.0 ± 7.6 nm	58.7 ± 8.2 nm
D10	91.1 ± 5.5 nm	67.2 ± 2.5 nm	91.7 ± 2.5 nm	107.6 ± 2.2 nm
D50	122.5 ± 1.8 nm	95.5 ± 4.8 nm	139.1 ± 6.8 nm	151.6 ± 5.7 nm
D90	179.9 ± 3.6 nm	162.4 ± 15.9 nm	311.8 ± 34.8 nm	240.9 ± 23.7 nm
Nanoparticles/mL	7.2 ± 0.79 × 10^12^	2.4 ± 0.2 × 10^12^	6.4 ± 0.5 × 10^12^	1.8 ± 0.2 × 10^13^

The mode, mean value, standard deviation of size, and concentration for each extracellular vesicle (EV) isolation from 200 uL of follicular and ampullary fluids. The value D50 represents the median size. Similarly, 90 percent of the distribution lies below the D90 value, and 10 percent of the population lies below the D10 value (mean +/− standard error). ODG UC: OptiPrep^TM^ density gradient ultracentrifugation. SEC: single-step size exclusion chromatography (qEV IZON™ single column).

**Table 2:** Effects of the experimental treatments on the in vitro embryo development.

Treatment ^1^	Fertilized Oocytes	Cleavage Rate (%) ^2^	Blastocyst Rate (%) ^3^
(1) Control	224	84.04 ± 1.15 ^a^	34.76 ± 1.69 ^c^
(2) Negative control	222	86.83 ± 1.15 ^a^	36.45 ± 1.69 ^bc^
(3) FF EVs SEC	216	87.52 ± 1.15 ^a^	35.85 ± 1.69 ^bc^
(4) AOF EVs SEC	221	87.85 ± 1.15 ^a^	39.79 ± 1.69 ^b^
(5) FF EVs + AOF EVs SEC	212	84.93 ± 1.15 ^a^	37.27 ± 1.69 ^bc^
(6) FF EVs ODG UC	218	86.72 ± 1.15 ^a^	45.41 ± 1.69 ^a^
(7) AOF EVs ODG UC	220	88.34 ± 1.15 ^a^	48.74 ± 1.69 ^a^
(8) FF EVs+ AOF EVs ODG UC	207	87.02 ± 1.15 ^a^	46.56 ± 1.69 ^a^

^1^ The treatments (eight groups) are defined in detail in the experimental design of the Materials and Methods and Figure 4. ^2^ The number of cleaved zygotes on day 2 out of the total number of fertilized oocytes (mean ± SE). ^3^ The number of blastocysts (day 8) out of the number of fertilized oocytes (mean ± SE). ^a, b, c^ Values in the same column with different superscripts differ significantly (*p* < 0.05). FF: follicular fluid. AOF: ampullary oviductal fluid.

**Table 3:** Effects of the experimental treatments on the differential apoptotic cell (AC) count measures of day 8 blastocysts.

Treatment ^1^	No. of Blast. ^2^	Cell Number			ACR (%) ^6^
		TCN ± SE ^3^	ICM ± SE ^4^	TE ± SE ^5^	
(1) Control	42	135.97 ±1.37 ^d^	36.97 ± 0.66 ^d^	99.00 ± 0.95 ^d^	12.49 ± 0.19 ^a^
(2) Negative control	46	138.23 ± 1.31 ^d^	38.71 ± 0.63 ^d^	99.52 ± 0.91 ^d^	11.59 ± 0.18 ^b^
(3) FF EVs SEC	48	149.02 ± 1.28 ^c^	41.75 ± 0.61 ^c^	107.27 ± 0.89 ^c^	8.06 ± 0.18 ^c^
(4) AOF EVs SEC	46	153.00 ± 1.31 ^c^	42.97 ± 0.63 ^bc^	110.02 ± 0.91 ^c^	7.85 ± 0.18 ^c^
(5) FF EVs + AOF EVs SEC	47	152.48 ± 1.30 ^c^	43.12 ± 0.62 ^bc^	109.36 ± 0.90 ^c^	6.55 ± 0.18 ^d^
(6) FF EVs ODG UC	51	161.94 ± 1.24 ^b^	44.39 ± 0.60 ^b^	117.54 ± 0.87 ^b^	6.18 ± 0.17 ^d^
(7) AOF EVs ODG UC	58	166.93 ± 1.17 ^ab^	46.91 ± 0.56 ^a^	120.01 ± 0.81 ^ab^	6.60 ± 0.16 ^d^
(8) FF EVs+ AOF EVs ODG UC	56	168.69 ± 1.19 ^a^	47.01 ± 0.57 ^a^	121.67 ± 0.83 ^a^	5.94 ± 0.16 ^d^

^1^ The treatments (eight groups) are defined in detail in the Experimental Design of the Materials and Methods. ^2^ Total number of day 8 blastocysts fixed for the differential cell count. ^3^ Total cell number (TCN): inner cell mass (ICM) plus trophectoderm (TE) (mean ± SE). ^4^ Inner cell mass (mean ± SE). ^5^ Trophectoderm (mean ± SE). ^6^ Apoptotic cell ratio (ACR) (mean ± SE). ^a, b, c, d^ Values in the same column with different superscripts differ significantly (*p* < 0.05).

## Data Availability

Not applicable.

## Abbreviations

EVs	Extracellular vesicles
FF	Follicular fluid
AOF	Ampullary oviduct fluid
ODG UC	OptiPrep™ density gradient ultracentrifugation
SEC	Size exclusion chromatography
ICM	Inner cell mass
TE	Trophectoderm
TCN	Total cell number
ACR	Apoptotic cell ratio
NTA	Nanoparticle tracking analysis
WB	Western blot
hpi	Hours post-insemination
